# Molecular mechanism of Mad2 conformational conversion promoted by the Mad2‐interaction motif of Cdc20

**DOI:** 10.1002/pro.70099

**Published:** 2025-03-27

**Authors:** Conny W. H. Yu, Elyse S. Fischer, Joe G. Greener, Jing Yang, Ziguo Zhang, Stefan M. V. Freund, David Barford

**Affiliations:** ^1^ MRC Laboratory of Molecular Biology Cambridge UK; ^2^ Present address: EMBL European Bioinformatics Institute Wellcome Genome Campus Hinxton CB10 1SD UK; ^3^ Present address: Monod Bio Seattle Washington US

**Keywords:** Cdc20, cell cycle, HORMA domain, Mad2, metamorphic proteins, molecular dynamic simulations, NMR, spindle assembly checkpoint

## Abstract

During mitosis, unattached kinetochores trigger the spindle assembly checkpoint by promoting the assembly of the mitotic checkpoint complex, a heterotetramer comprising Mad2, Cdc20, BubR1, and Bub3. Critical to this process is the kinetochore‐mediated catalysis of an intrinsically slow conformational conversion of Mad2 from an open (O‐Mad2) inactive state to a closed (C‐Mad2) active state bound to Cdc20. These Mad2 conformational changes involve substantial remodeling of the N‐terminal β1 strand and C‐terminal β7/β8 hairpin. In vitro, the Mad2‐interaction motif (MIM) of Cdc20 (Cdc20^MIM^) triggers the rapid conversion of O‐Mad2 to C‐Mad2, effectively removing the kinetic barrier for MCC assembly. How Cdc20^MIM^ directly induces Mad2 conversion remains unclear. In this study, we demonstrate that the Cdc20^MIM^‐binding site is inaccessible in O‐Mad2. Time‐resolved NMR and molecular dynamics simulations show how Mad2 conversion involves sequential conformational changes of flexible structural elements in O‐Mad2, orchestrated by Cdc20^MIM^. Conversion is initiated by the β7/β8 hairpin of O‐Mad2 transiently unfolding to expose a nascent Cdc20^MIM^‐binding site. Engagement of Cdc20^MIM^ to this site promotes the release of the β1 strand. We propose that initial conformational changes of the β7/β8 hairpin allow binding of Cdc20^MIM^ to a transient intermediate state of Mad2, thereby lowering the kinetic barrier to Mad2 conversion.

## 1 INTRODUCTION

During mitosis, accurate segregation of sister chromatids to daughter cells is critical to maintaining the faithful inheritance of genetic material and genomic stability. Chromatid segregation commences at the onset of anaphase when the cohesion between sister chromatids is released. This process is triggered only when all sister chromatid pairs have achieved successful bipolar attachment to the mitotic spindle, and tension is exerted at the microtubule‐kinetochore attachment site. Unattached kinetochores activate the spindle assembly checkpoint (SAC) (Rieder et al., 1995) inhibiting the anaphase‐promoting complex/cyclosome (APC/C) to delay anaphase onset. SAC signaling is mediated by the SAC effector, the mitotic checkpoint complex (MCC), a heterotetrameric complex consisting of Mad2, Cdc20, BubR1, and Bub3 (Chao et al., 2012; Sudakin et al., 2001), that binds to and inhibits APC/C^Cdc20^ (Alfieri et al., 2016; Izawa & Pines, 2015; Yamaguchi et al., 2016), reviewed in (Barford, 2020; Fischer, 2023; McAinsh & Kops, 2023; Musacchio, 2015; Watson et al., 2019).

**FIGURE 1:**
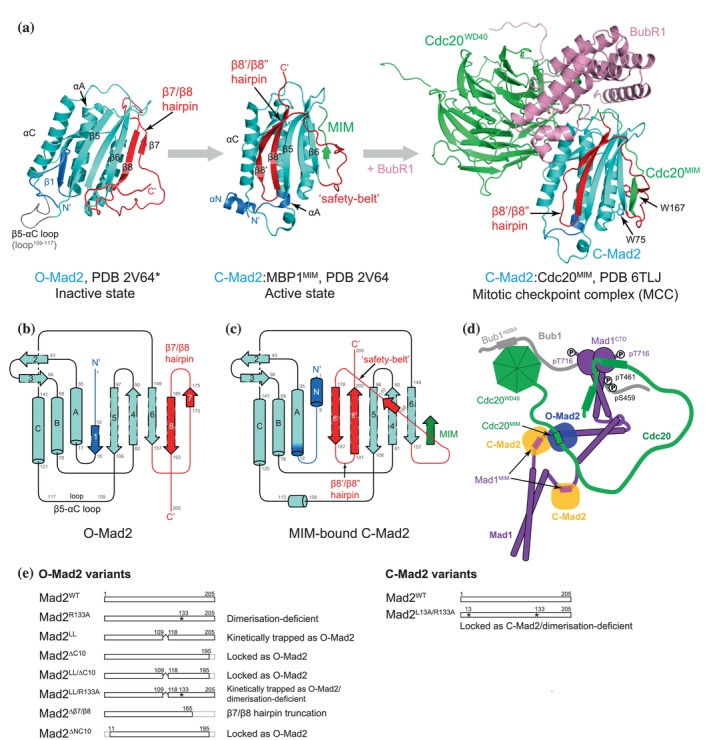
The conformational conversion of Mad2. (a) Representative structural models of Mad2 in different conformations. The O‐Mad2 structural model is based on the crystal structure of O‐Mad2^LL^ (PDB 2V64, chain D) (Mapelli et al., 2007) and the missing segments were built using Modeller (Webb & Sali, 2016). The C‐Mad2:MBP1^MIM^ structure is based on its crystal structure (PDB 2V64, chain A) (Mapelli et al., 2007). MBP1: Mad2‐binding peptide 1. The structure of C:Mad2:Cdc20^MIM^ in the BubR1:Cdc20:C‐Mad2 complex is extracted from the cryo‐EM structure of the APC/C:MCC complex (PDB 6TLJ) (Alfieri et al., 2016; Alfieri et al., 2020). The N‐terminal residues 1–15 are in blue and the C‐terminal residues 158–205 are in red to highlight the two segments that undergo significant conformational changes during the O‐ to C‐Mad2 conversion. (b, c) Secondary structure topology diagrams of O‐Mad2 (b) and C‐Mad2 (c). (d) Schematic of the MCC‐assembly scaffold. Mps1 kinase‐phosphorylated Bub1 recruits the Mad1:C‐Mad2 complex, O‐Mad2 (blue) is recruited through self‐dimerization with C‐Mad2 (orange), and Cdc20 interacts with Mps1‐phosphorylated Mad1^CTD^ (C‐terminal domain) and the ABBA motif of Bub1 (Fischer et al., 2022). Through the formation of a tripartite Bub1:Mad1:Cdc20 complex, the MCC‐assembly scaffold optimally positions Cdc20^MIM^ for its interaction with O‐Mad2 and triggers the conversion of O‐Mad2 to C‐Mad2. (e) Schematics of Mad2 variants used and discussed in this study. Mad2^R133A^, Mad2^LL^, Mad2^∆C10^, and Mad2^LL/∆C10^ were used for the backbone assignment of O‐Mad2.

MCC assembly requires the conversion of the metamorphic protein Mad2 from an inactive O‐Mad2 to an active C‐Mad2 state that has a high affinity for Cdc20 (De Antoni et al., 2005; Lad et al., 2009; Luo et al., 2000; Luo et al., 2002; Luo et al., 2004; Mapelli et al., 2007; Simonetta et al., 2009; Sironi et al., 2002) (Figure 1a–c). This C‐Mad2:Cdc20 complex spontaneously binds BubR1:Bub3 to generate the MCC (Chao et al., 2012; Faesen et al., 2017; Kulukian et al., 2009). Previous structural studies on these two states of Mad2, which have distinct secondary structure topologies, are summarized in Figure S1. O‐Mad2 and C‐Mad2 share a central core consisting of a three‐stranded antiparallel β sheet (β4‐β6), three α helices (αA‐αC), and a β hairpin (β2/β3) (cyan in Figure 1a–c). Whereas the central core of Mad2 remains mainly unchanged following the O‐ to C‐Mad2 conversion, the N‐ and C‐terminal segments undergo major remodeling. In O‐Mad2, the unstructured N‐terminus is followed by the five‐residue β1 strand, whereas the C‐terminus consists of the β7/β8 hairpin and an unstructured tail, with the β7/β8 hairpin pairing with β6 of the core β sheet. Upon conversion to C‐Mad2, the β7/β8 hairpin is displaced from β6 and repositioned, together with the C‐terminal tail, to form the β8′/β8″ hairpin that pairs with β5 on the opposite side of the β sheet. The β8′/β8″ hairpin substitutes for β1 of O‐Mad2, which is displaced to extend the N‐terminus of αA in C‐Mad2 (blue in Figure 1a–c). This rearrangement allows Cdc20^MIM^ to pair with β6 of the central β sheet. Importantly, the reconfigured C‐terminus of C‐Mad2 entraps Cdc20^MIM^ through a “safety‐belt” structure connecting β6 with β8′ (Sironi et al., 2002) (red in Figure 1a–c). This metamorphosis of Mad2 is a conserved feature of HORMA domain proteins (Aravind & Koonin, 1998; Fischer, 2023; Gu et al., 2022) in which a HORMA‐domain closure motif (here Cdc20^MIM^) is topologically linked within the closed state.

The intrinsic rate of the O‐Mad2 to C‐Mad2 conversion is extremely slow in solution (t_1/2_ of multiple hours) (Faesen et al., 2017; Lad et al., 2009; Luo et al., 2004; Simonetta et al., 2009), whereas in cells, the SAC response is established within minutes (Clute & Pines, 1999; Dick & Gerlich, 2013; Meraldi et al., 2004). Unattached kinetochores activate the SAC signal by catalyzing the formation of the MCC (Kulukian et al., 2009), in which the key catalysts of MCC assembly are the protein kinase Mps1, the Mad1:C‐Mad2 heterotetramer, and Bub1 (Faesen et al., 2017; Ji et al., 2017; Piano et al., 2021; Sethi et al., 2024). These catalysts generate and contribute to an MCC‐assembly scaffold that accelerates the formation of the C‐Mad2:Cdc20 complex (Chen et al., 2023; Fischer et al., 2021; Fischer et al., 2022; Ji et al., 2017; Lara‐Gonzalez et al., 2021; Piano et al., 2021). Mps1 activity, stimulated by the Ndc80 complex at unattached kinetochores (Hiruma et al., 2015; Ji et al., 2015), phosphorylates Knl1 and Bub1, thereby promoting Bub1 interactions with Mad1 and Cdc20. This scaffold both promotes the accessibility of Cdc20^MIM^ (through a proposed conformational change of Cdc20 (Han et al., 2013; Lara‐Gonzalez et al., 2021; Piano et al., 2021; Zhang & Lees, 2001) and juxtaposes Cdc20^MIM^ adjacent to its cryptic binding site on an O‐Mad2 molecule interacting with C‐Mad2 of the Mad1:C‐Mad2 complex (Chen et al., 2023; Fischer et al., 2022; Lara‐Gonzalez et al., 2021; Piano et al., 2021) (Figure 1d). Accordingly, this catalyzes the formation of C‐Mad2:Cdc20 (Piano et al., 2021). In support of this model, NMR experiments revealed that high concentrations of a peptide modeled on Cdc20^MIM^ (200 μM, in a two‐fold molar excess over O‐Mad2) accelerated by at least 50‐fold the entrapment of Cdc20^MIM^ by O‐Mad2 to form C‐Mad2:Cdc20^MIM^, as compared with the conversion rate of O‐Mad2 to “empty” C‐Mad2 in the absence of MIM peptide (Fischer et al., 2022).

Juxtaposing the accessible Cdc20^MIM^ adjacent to O‐Mad2 by the MCC‐assembly scaffold involves the dimerization of an O‐Mad2 molecule with Mad1‐bound C‐Mad2 (Chen et al., 2023; Fischer et al., 2022; Lara‐Gonzalez et al., 2021; Piano et al., 2021). C‐Mad2 binds to Cdc20^MIM^ and the MIM of Mad1 (Mad1^MIM^) through comparable mechanisms (Luo et al., 2002; Luo et al., 2004; Mapelli et al., 2007; Sironi et al., 2002). In vivo studies demonstrated that disrupting the C‐Mad2:O‐Mad2 interaction abrogates SAC signaling (De Antoni et al., 2005; Mapelli et al., 2007; Nezi et al., 2006). Additionally, the template model posits that C‐Mad2:Mad1 contributes directly to catalyzing MCC assembly by acting as a structural template for the conversion of O‐Mad2 to C‐Mad2 (De Antoni et al., 2005; Mapelli et al., 2007). This model is consistent with in vitro biochemical data showing that the O‐Mad2 to C‐Mad2 conversion rate is modestly enhanced (3 to 4 fold) by dimerization with C‐Mad2 (Lad et al., 2009; Simonetta et al., 2009), and that purified chromosomes enhance the formation of the MCC through a Mad2 template‐dependent manner (Kulukian et al., 2009). However, in vitro, the rate enhancement contributed by O‐Mad2:C‐Mad2 dimerization alone is substantially lower than the rate of MCC assembly in cells during an active SAC, consistent with the observations discussed above that the other MCC assembly catalysts are critical to accelerating MCC formation (Faesen et al., 2017; Ji et al., 2017; Piano et al., 2021).

Despite decades of structural studies on Mad2 and recent progress in elucidating the molecular mechanisms underlying catalytic MCC assembly, our understanding of the molecular mechanisms of the O‐Mad2 to C‐Mad2 conversion and how this is promoted by the MIM remains incomplete. In this study, we combined time‐resolved NMR and molecular dynamics (MD) simulations to investigate the dynamics of structural elements in O‐Mad2. We provide evidence that the β7/β8 hairpin is flexible and that the inaccessible Cdc20^MIM^‐binding site on O‐Mad2 becomes exposed upon the transient unfolding of the β7/β8 hairpin. By tracing the initial conversion rates of individual residues in O‐Mad2, we demonstrate that Cdc20^MIM^ promotes the remodeling of the β1 strand, likely by facilitating its release from the core. Our data also further indicate the existence of intermediate states during the O‐Mad2 to C‐Mad2 conversion. We propose that the conversion of O‐Mad2 to C‐Mad2 in the presence of Cdc20^MIM^ is initiated by O‐Mad2 spontaneously unfolding to expose the Cdc20^MIM^ binding site. This allows binding of Cdc20^MIM^ to an intermediate state of Mad2, stabilizing this state, thereby lowering the kinetic barrier of O‐Mad2 conversion to C‐Mad2:Cdc20^MIM^.

## 2 RESULTS

### 2.1 NMR fingerprinting of Mad2

We used time‐resolved solution NMR to investigate the dynamic properties of the O‐Mad2 to C‐Mad2 conversion. We collected ^1^H, ^15^N 2D HSQC spectra of Mad2 in different conformational states using different Mad2 variants (Figure 1e) and in the presence of a variety of MIM ligands. To gain structural insights on full‐length Mad2, using the dimerization‐deficient mutant Mad2^R133A^ (Sironi et al., 2002), we determined a near‐complete assignment of Mad2 in its open state (O‐Mad2), “empty” closed state (C‐Mad2), and “bound” closed state (C‐Mad2:Cdc20^MIM^). The assignments are described in Figure S2a–c and in the Methods.

**FIGURE 2:**
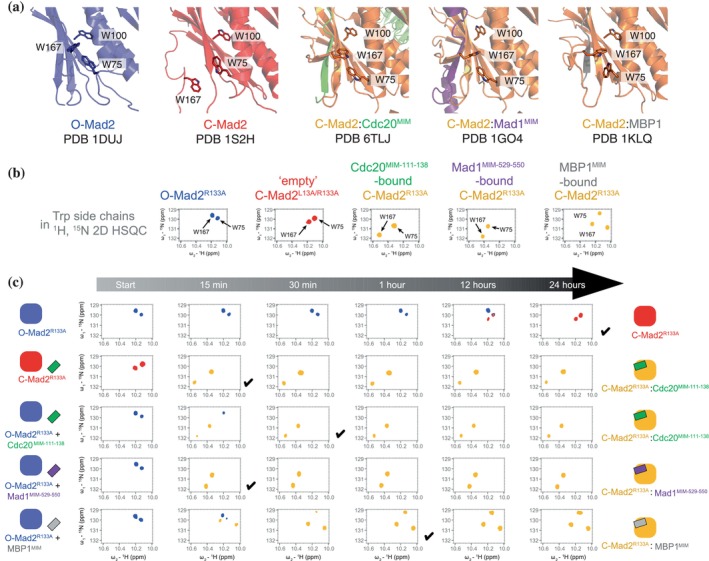
NMR fingerprinting of Mad2. (a) Structural orientations of Trp75 (on the αB helix) and Trp167 (on the “safety‐belt”) in Mad2 produce distinct indole NH resonances. Trp167 is displaced during conversion, while Trp75, near the MIM‐binding site, shows significant perturbations with different MIM ligands. (b) Indole NH resonances of Trp75 and Trp167 exhibit distinct chemical shifts in different Mad2 conformations. NMR spectra for O‐Mad2^R133A^ and “empty” C‐Mad2^L13A/R133A^ and C‐Mad2^R133A^:Cdc20^MIM‐111‐138^ are from Fischer et al. (2022) (c) Mad2 conversion rates vary with different MIM peptides. Conversion was traced over 24 h at 25°C using the Trp75 and Trp167 side‐chain resonances as reporters. MIM peptides were added at two‐fold molar excess to Mad2 (100 μM). Each spectrum required 12 min of acquisition time, therefore the 15 min time‐points represent the earliest spectra in the time‐resolved series. The schematics show the conformation of Mad2 as indicated by the tryptophan side‐chain resonances. The full time‐resolved spectra are shown in Figures S4 and S5. A black tick marks when conversion is considered complete. NMR data for O‐Mad2^R133A^ with and without Cdc20^MIM‐111‐138^ shown are from Fischer et al. (2022). In the case of full‐length Cdc20, the MIM sequence is sequestered by an auto‐inhibitory intramolecular interaction within Cdc20 itself, which prevents it from being readily accessible for binding to C‐Mad2 (Piano et al., 2021). Conversely, isolated MIM peptides lack this auto‐inhibitory mechanism, allowing them to bind directly to C‐Mad2 without obstruction.

In the absence of MIM, O‐Mad2 slowly converts to “empty” C‐Mad2 (Fischer et al., 2022), which structurally resembles the MIM‐bound C‐Mad2 (Figure S3a). Despite these structural similarities, the NMR fingerprints for “empty” C‐Mad2 and the C‐Mad2:Cdc20^MIM^ complex show large chemical shift differences (Figure S2d) (Fischer et al., 2022). These differences arise from the rearrangement of aromatic residues whose ring currents influence the nuclear environments of backbone amides in their close proximity (Figure 2a). Likewise, despite the structural similarities of the ligand‐bound C‐Mad2 complexes, their 2D spectra are characteristic of specific MIM peptides (Figure S2e). Differences were observed for the indole NH resonances of Trp75 and Trp167 (Figure 2b). In particular, for Trp75, which is situated close to the MIM‐binding site, significant perturbation was observed with the bound ligand (Figure 2a,b). The indole NH resonances of Mad2 tryptophan side chains were therefore used to identify the conformational state of the Mad2 MIM‐binding site and its bound ligand.

We then investigated the secondary structures of the two Mad2 conformational states using their secondary chemical shifts. Secondary chemical shifts report deviations of the experimental Cα and Cβ chemical shifts from the theoretical values for a random coil (Spera & Bax, 1991). A positive value indicates helical propensity, whereas a negative value indicates β‐strand propensity. Overall, our secondary chemical shifts correlate well with the secondary structures of experimentally determined structures (Figure S3). For O‐Mad2, we observed no secondary structure for the N‐ and C‐terminal 10 residues (Figure S3b). In solution, the N‐terminal 10 residues did not adopt a stable αN helix in either “empty” C‐Mad2 or C‐Mad2:Cdc20^MIM^ (Figure S3c,d). A short αN helix observed in the crystal structure of C‐Mad2:MBP1^MIM^ (MBP1^MIM^:MIM of Mad2‐binding peptide1 (Luo et al., 2002) dimerized with O‐Mad2 (Mapelli et al., 2007) is absent in the cryo‐EM structure of the APC/C:MCC complex (Alfieri et al., 2016; Alfieri et al., 2020) and any other crystal structure of full‐length C‐Mad2 (Figure S1) (Yang et al., 2008).

### 2.2 The MIM‐binding site is inaccessible in O‐Mad2

**FIGURE 3:**
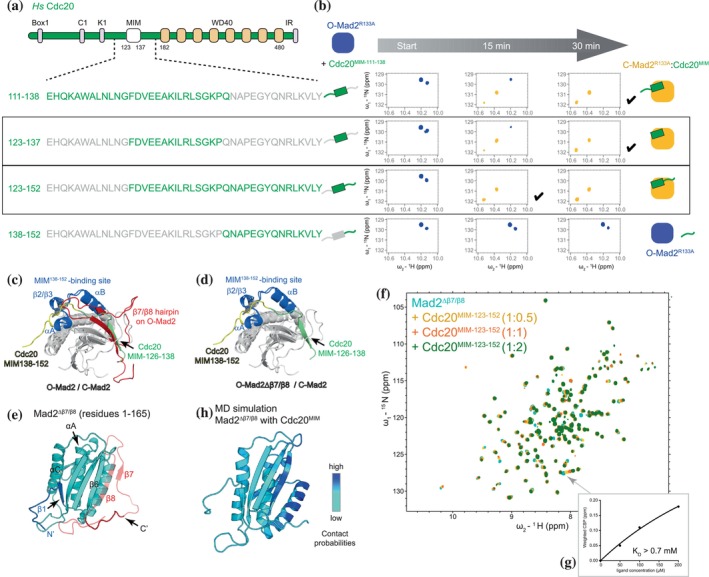
Mad2 conversion rates with different Cdc20^MIM^ peptides. (a) Schematic of human Cdc20. Box1: Box1 motif; C1: C‐box motif; K1: KEN box motif; MIM: Mad2‐interacting motif, residues 123–137; WD40: WD40 domain, residues 182–480; IR: IR tail. (b) Mad2 conversion in the presence of different Cdc20^MIM^ peptides. Mad2 conversion was traced at 25°C and the changes in the first 30 min are shown. The schematics illustrate the lengths of different MIM peptides, and the full spectra are shown in Figures S4a and S8. A black tick indicates where conversion is considered complete. NMR data for O‐Mad2^R133A^ with Cdc20^MIM‐111‐138^ shown for comparison are from Fischer et al. (2022). (c) The Cdc20^MIM‐138‐152^‐binding site was identified by comparing the spectra of C‐Mad2:Cdc20^MIM‐123‐137^ with C‐Mad2:Cdc20^MIM‐123‐152^ (boxed in b) and analyzing their CSPs (Figure S8e,f). The Cdc20^MIM‐138‐152^‐binding site (blue) is mapped onto the structure of C‐Mad2:Cdc20 from the cryo‐EM structure of the APC/C:MCC complex (PDB 6TLJ) (Alfieri et al., 2016, Alfieri et al., 2020). The O‐Mad2 structural model is superimposed onto the C‐Mad2 structure for comparison. Cdc20 residues 138–152 are in yellow and residues 126–138 are in green. The β7/β8 hairpin in O‐Mad2 (red) blocks both the binding sites for Cdc20 residues 126–138 and residues 138–152. (d) As in c except the β7/β8 hairpin in O‐Mad2 is removed to expose the Cdc20 binding sites. (e) Model of the Mad2^∆β7/β8^ variant (residues 1–165; Δ166‐205) to test Cdc20^MIM^‐binding. The truncated residues 166–205 are shown with transparency. (f) Cdc20^MIM‐123‐152^ was titrated into Mad2^∆β7/β8^ and its binding was analyzed by NMR. (g) Resonance with the largest CSP in f (marked by an arrow) was used to estimate the binding affinity. (h) Mad2^∆β7/β8^ color‐coded according to MIM contact probabilities from MD simulations totaling 12 μs of Mad2^∆β7/β8^ in the presence of the MIM peptide.

To understand whether Mad2 conversion times are modulated by different C‐Mad2 ligands, we used time‐resolved NMR to determine the conversion time of 100 μM O‐Mad2^R133A^ at 25°C. The indole NH resonances of Trp75 and Trp167 were used as reporters (Figure 2b). We previously showed that in the presence of Cdc20^MIM‐111‐138^ (Cdc20^MIM^ peptide spanning Cdc20 residues 111–138), complete Mad2 conversion occurred in less than 30 min (Fischer et al., 2022) (Figures 2c, 3a and S4a). In contrast, conversion took ~24 h in the absence of Cdc20^MIM‐111‐138^ peptide (Fischer et al., 2022) (Figures 2c and S4b). Thus, the MIM peptide accelerates the O‐Mad2 to C‐Mad2 conversion by nearly 50‐fold, comparable to the 35‐fold acceleration of MCC assembly in the presence of full‐length Cdc20 and the catalytic scaffold (Mad1:C‐Mad2, Bub1:Bub3, Mps1) (Piano et al., 2021). These data support the idea that an essential role of the MCC assembly catalysts is to present Cdc20^MIM^ at high effective concentration and optimal orientation proximal to the cryptic Cdc20^MIM^‐binding site in O‐Mad2. Other MIM peptides also accelerated the O‐Mad2 to C‐Mad2 conversion. In the presence of Mad1^MIM‐529‐550^ (Mad1 residues 529–550), conversion reached completion within 15 min (i.e., within the acquisition time of an individual HSQC spectrum), and 60 min in the presence of MBP1^MIM^, a phage‐display MIM peptide selected for its enhanced C‐Mad2 affinity (Luo et al., 2002) (Figures 2c and S5a,b). Our finding that Mad1^MIM‐529‐550^ induced the O‐Mad2 to C‐Mad2 conversion agrees with an earlier NMR study showing that Mad1 residues 540–551 facilitate this conversion (Luo et al., 2004). We observed that “empty” C‐Mad2 rapidly adopted a “bound” C‐Mad2:Cdc20^MIM^ conformation on the addition of Cdc20^MIM^ (Figures 2c and S4c), indicating that the MIM‐binding site is easily accessible in “empty” C‐Mad2 for MIM peptides, consistent with prior observations that “empty” C‐Mad2 readily binds Cdc20^MIM^ when presented as short peptides (Lad et al., 2009; Piano et al., 2021). In contrast, “empty” C‐Mad2 does not readily associate with full‐length Cdc20, as an auto‐inhibitory mechanism sequesters the MIM sequence through intramolecular interactions (Piano et al., 2021). In “empty” C‐Mad2, the β7/β8 hairpin is displaced to expose the β6 strand for pairing with the MIM peptide (Luo et al., 2004) (Figure S3a). O‐Mad2 that is unable to convert to C‐Mad2 does not bind Cdc20^MIM^, as determined from NMR spectra when Cdc20^MIM^ was titrated into Mad2^∆C10^ (Figures S6a–c and S7a), a Mad2 variant trapped in the open state (De Antoni et al., 2005), consistent with previous studies that Mad2^∆C10^ does not interact with full‐length Cdc20 (Fang et al., 1998; Luo et al., 2000; Luo et al., 2004).

### 2.3 An extended binding site for Cdc20^MIM^ involving linker residues 138–152

Cdc20^MIM‐111‐138^ had previously been used to study the O‐Mad2 to C‐Mad2 conversion (Fischer et al., 2022) (Figures 2c and S4a). However, it remained unclear whether Cdc20‐binding sites for Mad2 are limited to this region, given the extended linker connecting the MIM and WD40 domain of Cdc20 (residues 139–152) (Figure 3a) that might interact with Mad2. Furthermore, Cdc20 residues N‐terminal of Glu126 were not resolved in the cryo‐EM structure of the human APC/C:MCC complex (Alfieri et al., 2016; Alfieri et al., 2020), and it was ambiguous if these residues contribute to Mad2 binding and conversion. To identify potential additional Mad2‐binding residues on Cdc20 and the corresponding Cdc20‐binding sites on Mad2, we designed different length Cdc20^MIM^ peptides and compared their effects on Mad2‐conversion rates by NMR (Figures 3a,b and S8). Cdc20^MIM^ spanning residues 123–137 (Cdc20^MIM‐123‐137^) triggered Mad2 conversion at a similar rate as Cdc20^MIM‐111‐138^ (Figures 3b, S4a and S8a). Moreover, there were no major chemical shift differences between the spectra of C‐Mad2:Cdc20^MIM‐123‐137^ and C‐Mad2:Cdc20^MIM‐111‐138^ (Figure S8b), indicating that residues 111–122 are not involved in either inducing the O‐Mad2 to C‐Mad2 conversion or interacting with C‐Mad2. In contrast, Cdc20^MIM‐123‐152^ that incorporates the PEG motif (residues 138–144) was more efficient at triggering Mad2 conversion, with conversion complete within 15 min (Figures 3b and S8c). This aligns with the observation that removal of the PEG motif reduces the catalytic incorporation of Cdc20 into the MCC (Piano et al., 2021). However, Cdc20 residues 138–152 failed to induce Mad2 conversion after two hours, and therefore alone are insufficient to bind Mad2 (Figures 3b and S8d).

Comparing the spectra of C‐Mad2:Cdc20^MIM‐123‐152^ and C‐Mad2:Cdc20^MIM‐123‐137^ (Figure S8e) identified an additional Cdc20‐binding site on Mad2, as revealed by chemical shift perturbations (CSPs) dependent on the additional Cdc20 residues 138–152 (Figures 3c and S8f). This Cdc20^MIM‐138‐152^‐binding site comprises residues of the αA‐β2 and β3‐αB loops of Mad2. In the cryo‐EM map of the APC/C:MCC complex (Alfieri et al., 2016; Alfieri et al., 2020), residues 134–143 of Cdc20 are located in close proximity to this site. However, the resolution in this region of the cryo‐EM map limited our analysis of the interaction between Cdc20^MIM‐138‐152^ and C‐Mad2. The relatively small CSPs and lack of structural information on Cdc20^MIM‐138‐152^ suggested that residues 138–152 of Cdc20 are only weakly associated with C‐Mad2. Similar to the major MIM‐binding site involving β6, the putative Cdc20^MIM‐138‐152^‐binding site is blocked by the β7/β8 hairpin (colored red in Figure 3c). Overall, these results indicate that Cdc20 residues 123–138 constitute the core of Cdc20^MIM^, consistent with the APC/C:MCC cryo‐EM structure (Alfieri et al., 2016; Alfieri et al., 2020), with residues 138–152 contributing to affinity through avidity effects.

We reasoned that MIM binding to Mad2 is blocked until the β7/β8 hairpin is displaced (Figure 3c,d). To test this hypothesis, we generated a Mad2 variant with a deleted β7/β8 hairpin (Mad2^∆β7/β8^: residues 1–165) (Figure 3e). Mad2^∆β7/β8^ adopted a stable globular fold, and due to the absence of the C‐terminal residues, it was unable to adopt a closed conformation (Figure 3f). CSPs induced by the addition of Cdc20^MIM‐123‐152^ indicated a weak interaction between Cdc20^MIM‐123‐152^ and Mad2^∆β7/β8^, with an estimated K_D_ of >0.7 mM (Figure 3f,g). This is in agreement with a previous study that showed a weak interaction between Mad2^∆β7/β8^ and MBP1^MIM^ (Yang et al., 2008). We performed molecular dynamic simulations to investigate the MIM‐binding site on Mad2^∆β7/β8^, calculating simulations of Mad2^∆β7/β8^ in the presence of the MIM peptide. MIM‐contact probabilities mapped onto the Mad2^∆β7/β8^ structure are shown in Figure 3h. Binding of MIM is localized to the αB‐helix and β6, a finding that supports the idea that MIM binds to the exposed β6 strand and likely contributes to displacing β7/β8.

### 2.4 A model for how unfolding the β7/β8 hairpin would expose the MIM‐binding site

**FIGURE 4:**
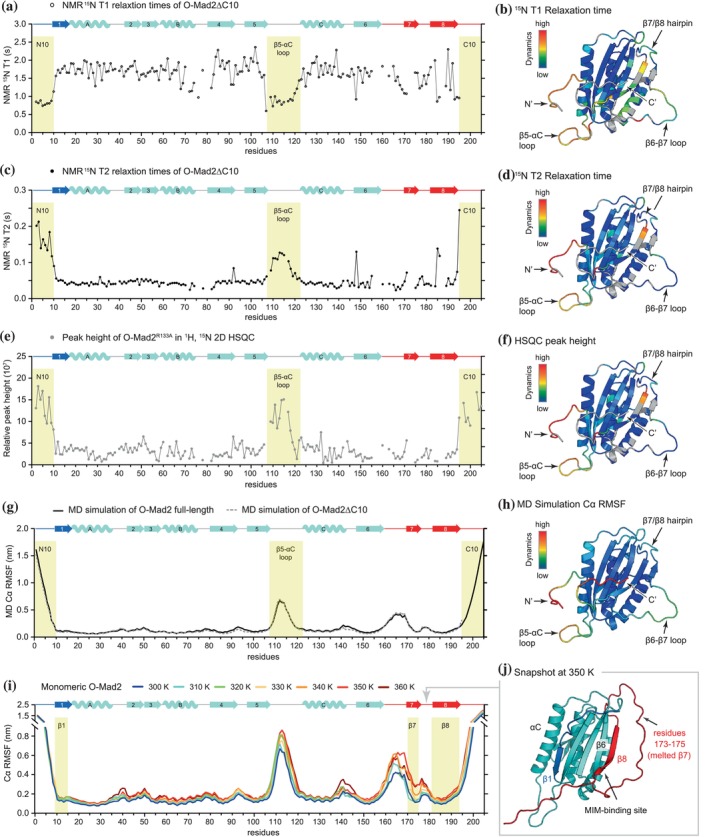
The C‐terminal β7/β8 hairpin may transiently unfold to expose the MIM‐binding site. NMR and MD simulations were used to study the dynamics of O‐Mad2. A total of 5.4 μs of simulations were carried out for each variant, using six independent simulations of 0.9 μs. RMSD plots for all MD simulations are shown in Figure S9. (a) ^15^N T1 relaxation times were measured at 298 K. Regions that are highly dynamic are highlighted in yellow. (b) Color‐ramp of T1 relaxation data mapped on to the O‐Mad2 structural model. (c) ^15^N T2 relaxation times were measured at 298 K. (d) Color‐ramp of T2 relaxation data mapped on to the O‐Mad2 structural model. (e) HSQC peak heights for the O‐Mad2^R133A^ mutant (from Figure S2a). (f) Color‐ramp of HSQC peak heights data mapped on to the O‐Mad2 structural model. (g) Cα RMSF were determined from MD simulations at 300 K for full‐length O‐Mad2 (black lines) and O‐Mad2^∆C10^ (dotted gray lines). Regions that are highly dynamic are highlighted in yellow. (h) Color‐ramp of dynamics data from MD simulations mapped on to the O‐Mad2 structural model. (i) Cα RMSF determined from MD simulations at 300–360 K for monomeric O‐Mad2. (j) A snapshot of the O‐Mad2 simulation at 350 K acquired after 0.6 μs of simulation.

How Cdc20^MIM^ induces the O‐Mad2 to C‐Mad2 conversion when the Cdc20‐binding sites are not accessible in O‐Mad2 is unknown. Due to the dynamic properties of O‐Mad2, we hypothesized that MIM‐binding sites might be transiently exposed on the displacement of the β7/β8 hairpin. To assess this, we analyzed the backbone dynamics of O‐Mad2 using a combination of NMR relaxation experiments and MD simulations. NMR ^15^N relaxation (T1 and T2) data provide information on backbone mobility on the picosecond to nanosecond timescale and are therefore a reliable diagnostic of protein dynamics. We measured the ^15^N T1 and T2 relaxation times for O‐Mad2^∆C10^ but were unable to collect equivalent data for O‐Mad2^R133A^ because this variant slowly converted to C‐Mad2 during data acquisition. The HSQC spectrum for O‐Mad2^∆C10^ overlaps with those of O‐Mad2^WT^ and O‐Mad2^R133A^, confirming that these variants share a similar structural conformation (Figure S6a). Assignment of the backbone amide resonances in O‐Mad2^∆C10^ revealed missing signals for the β7/β8 hairpin region (residues 160–190). This was most likely due to exchange broadening, indicative of slow conformational averaging of the β7/β8 hairpin. The T1 and T2 relaxation times for O‐Mad2^∆C10^ are shown in Figure 4a,c, respectively, with T1 and T2 relaxation times, indicative of dynamics, visualized as color gradients on the O‐Mad2 structural model (Figure 4b,d). The longest T2 relaxation times map to the N‐terminus, β5‐αC loop, and the tip of the β7/β8 hairpin, indicating that these regions are highly flexible on a picosecond timescale and are predominantly unstructured. This is also supported by ^15^N{^1^H}‐heteronuclear NOE experiment that samples backbone dynamics (Figure S6d,e). On the other hand, the exchange broadening for the β7/β8 hairpin region suggests that under these conditions, a slow interconversion between multiple partly structured states occurs. The assignment of flexible and dynamic regions of O‐Mad2^∆C10^ to the N‐terminus, β5‐αC loop, and β7/β8 hairpin from T2 relaxation data correlates with the assignment of flexible regions of O‐Mad2^∆C10^ based on the T1 relaxation data (Figure 4a,b). While we were unable to collect equivalent data for full‐length O‐Mad2^R133A^ as this variant slowly undergoes conversion, its 2D HSQC spectra provide a glimpse of the dynamics of the full‐length protein (Figures 4e,f and S2a). In agreement with the dynamics data of O‐Mad2^∆C10^, the N‐terminus and β5‐αC loop in full‐length O‐Mad2^R133A^ are highly flexible, and the C‐terminal residues are also highly dynamic. Unfortunately, dynamics data at the β7/β8 hairpin remain incomplete due to exchange broadening in this region.

In our MD simulations, we performed a total of 5.4 μs of simulations at 300 K on both full‐length O‐Mad2 and O‐Mad2^∆C10^. To assess residue flexibility, we analyzed the root mean square fluctuation (Cα RMSF) of Cα atoms, which measures the magnitude of movement for each Cα atom over the simulation (Figure 4h). RMSD plots are shown in Figure S9. Given the timescale of the O‐Mad2 to C‐Mad2 conversion (~24 h at 25°C, Figure 2d), these simulations could not capture the entire open‐to‐closed conversion. However, the Cα RMSF for O‐Mad2 and O‐Mad2^∆C10^ are highly similar (Figure 4g), indicating that the C‐terminal deletion does not significantly affect the backbone dynamics of O‐Mad2.

Our T1/T2 relaxation and MD simulation data indicated that O‐Mad2 consists of a stable protein core with flexible regions comprising the 10 residues at both N‐ and C‐termini, the β5‐αC loop, and residues 160–190 (β6/β7 loop and β7/β8 hairpin) (Figure 4a–d,g,h). A dynamic N‐terminus (residues 1–10) agrees with our observations that there was no residual helical propensity for αN (Figure S3b). There is a sharp drop in backbone dynamics at residue 11 of the β1 strand (residues 11–15), suggesting that the β1 strand is stably bound to the core of O‐Mad2. MD simulations further indicated a highly flexible β6/β7 loop connecting the β6 strand with the β7/β8 hairpin, with an overall increase in dynamics in this region but a marked dip at the β8 strand (Figure 4h). In the O‐Mad2^∆C10^ variant, where the C‐terminal 10 residues were deleted, T1/T2 relaxation NMR data could not assess the dynamics of these residues. However, the Cα RMSF data indicate a highly dynamic C‐terminus in O‐Mad2, including residues 191–200, which remodel to become the β8″ strand in C‐Mad2 (Figure 1b,c). This agrees with the HSQC peak height analysis of full‐length O‐Mad2^R133A^ (Figure 4e,f).

Upon the O‐Mad2 to C‐Mad2 conversion, two structural elements are released from the core of O‐Mad2: the N‐terminal β1 strand and the C‐terminal β7/β8 hairpin (Figure 1a). Both are directly connected to highly flexible termini (Figure 4c,d), and we hypothesized that either element might be transiently displaced to initiate conversion. We reasoned that the melting of the short β7 strand (residues 173–175) is likely the first event in Mad2 conversion as β7 is held in place by only three main‐chain hydrogen bonds. To test this hypothesis, we performed MD simulations at increasing temperatures to investigate which structural elements are least stable.

We performed MD simulations on full‐length O‐Mad2, using a temperature range from 300 to 360 K (Figures 4i and S9). A total of 5.4 μs of simulations were performed at each temperature. The protein core of O‐Mad2 remained stable from 300 to 340 K, consistent with the experimentally determined melting temperature of 342 K (Zhao et al., 2016). At 350 K, the MD simulations suggest that β7 dissociates from the core, resulting in an increased Cα RMSF (Figure 4i,j). The C‐terminal half of β8, which pairs with β6, was also found to be dynamic in all simulations, occasionally peeling off from β6 at 350 K (Figure 4i). This would expose the binding site for MIM on the C‐terminal half of β6. Meanwhile, β1 and the N‐terminal half of β8 remained bound to the core even at the high simulated temperature of 360 K (Figure 4i). The transient unfolding of the C‐terminal β7/β8 hairpin would allow O‐Mad2 to bind Cdc20^MIM^; however, as Cdc20^MIM^ only weakly associates with Mad2^∆β7/β8^ (Figure 3f,g), the transient exposure of the MIM‐binding site alone is likely not sufficient to secure Cdc20^MIM^ binding. A complete displacement of the β7/β8 hairpin, together with the β1 strand, is necessary to entrap Cdc20^MIM^ through the formation of the “safety‐belt.” A flexible β7/β8 hairpin contrasting with a more rigid β1 strand is consistent with prior hydrogen deuterium exchange data revealing fast amide proton exchange for β8 and slow exchange for β1 (Yang et al., 2007). We also note that the C‐terminus of the αC helix became more flexible at 360 K.

In conclusion, our NMR data and MD simulations are consistent with a model that a flexible β7/β8 hairpin would expose the Cdc20 MIM‐binding site on Mad2. However, direct observation of the spontaneous unfolding of the β7/β8 hairpin is lacking.

### 2.5 The dynamic β5‐αC loop of O‐Mad2 modulates the O‐Mad2 to C‐Mad2 conversion

**FIGURE 5:**
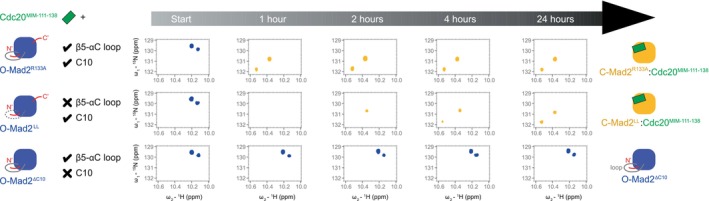
The dynamic β5‐αC loop and C‐terminal residues in O‐Mad2 modulate Mad2 conversion. The O‐ to C‐Mad2 conversion is slowed when the highly dynamic β5‐αC loop is truncated (O‐Mad2^LL^) and eliminated in the absence of the C‐terminal ten residues (O‐Mad2^∆C10^). Full spectra for O‐Mad2^LL^ and O‐Mad2^ΔC10^ are shown in Figure S7. Using the side chain resonances of Trp75 and Trp167 as reporters, Mad2 conversion was traced in a 24 h time course at 25°C. Cdc20^MIM‐111‐138^ peptides were added at two‐fold molar excess to Mad2 (100 μM). Severe line broadening was observed during the conversion of O‐Mad2^LL^, most likely due to dimerization of O‐Mad2^LL^ with the newly converted C‐Mad2^LL^. NMR data for O‐Mad2^R133A^ with Cdc20^MIM‐111‐138^ shown for comparison are from Fischer et al. (2022).

To understand the contributions of the dynamic β5‐αC loop in the O‐Mad2 to C‐Mad2 conversion, we examined the loop‐less Mad2 mutant (Mad2^LL^) in which the β5‐αC loop is truncated (Mapelli et al., 2007). This truncation creates a kinetic barrier to the remodeling of the β1 strand, severely reducing rates of conversion (Mapelli et al., 2007). We observed no binding of Cdc20^MIM‐111‐138^ to Mad2^LL^ in the NMR spectra collected early in the time course (Figure S6f). However, the spectra revealed a gradual, time‐dependent change, indicating a conformational transition of O‐Mad2^LL^ to C‐Mad2^LL^ (Figures 5 and S7b). The final spectrum of C‐Mad2^LL^:Cdc20^MIM^ closely matched that of full‐length C‐Mad2:Cdc20^MIM^ (Figure S7c,d). In the presence of Cdc20^MIM‐111‐138^, the conversion of O‐Mad2^LL^ to C‐Mad2^LL^:Cdc20^MIM^ took ~250 min, 8‐times slower than the conversion of O‐Mad2^R133A^ with Cdc20^MIM‐111‐138^ (Figure 5). Given that the slow conversion of O‐Mad2^LL^ to C‐Mad2^LL^:Cdc20^MIM^ is caused by the shorter β5‐αC loop posing a kinetic barrier to β1 remodeling (Mapelli et al., 2007), we conclude that Mad2 conversion is dependent on β1 remodeling.

### 2.6 O‐Mad2 retains its dynamic and structural properties in the O‐Mad2:C‐Mad2 dimer

**FIGURE 6:**
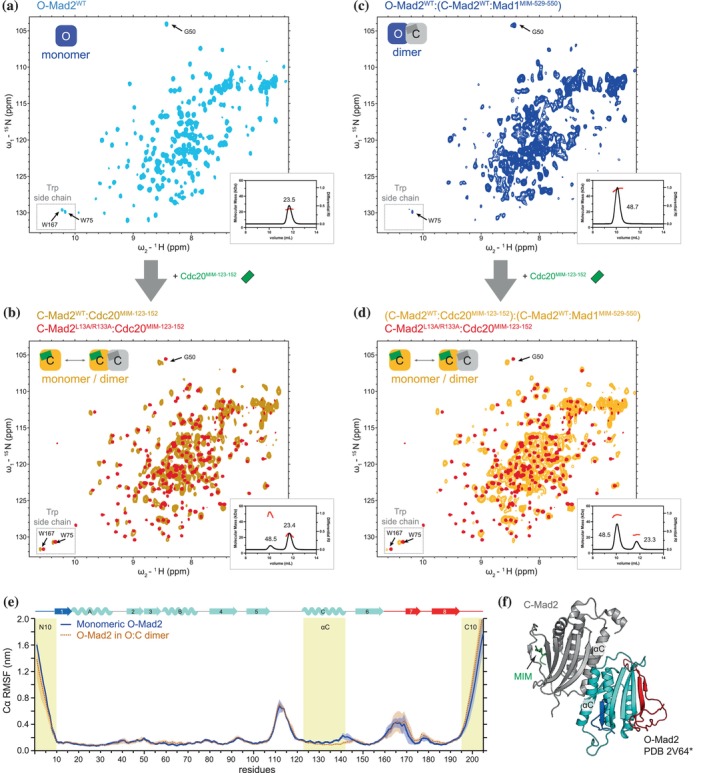
O‐Mad2 retains its dynamic and structural properties in the O‐Mad2:C‐Mad2 dimer. (a, c) HSQC spectra of 300 μM wild‐type O‐Mad2^WT^ (a, light blue) and O‐Mad2^WT^:(C‐Mad2^WT^:Mad1^MIM^) (c, blue). The schematics show the conformation of Mad2 as indicated by the tryptophan side‐chain resonances and their oligomeric states as determined by SEC‐MALS (insets). The SEC peaks are labeled with their molecular masses (kDa), where the theoretical molecular masses for monomeric and dimeric Mad2 are 23.5 and 47.0 kDa, respectively. For the dimeric samples, only one of the monomers was isotopically labeled and observable by NMR. Distinct chemical shift differences were also observed for Gly50 in O‐Mad2 and C‐Mad2, and its resonances are marked in the spectra. Cdc20^MIM‐123‐152^ was added at two‐fold molar excess to O‐Mad2^WT^ (a), and O‐Mad2^WT^:(C‐Mad2^WT^:Mad1^MIM^) (c). (b, d) This generated C‐Mad2^WT^‐Cdc20^MIM^ (b, brown), and (C‐Mad2^WT^:Cdc20^MIM^):(C‐Mad2^WT^:Mad1^MIM^) (d, yellow), respectively. Their spectra are overlaid with that of C‐Mad2^L13A/R133A^:Cdc20^MIM^ (red) for comparison. (e) Cα RMSF were determined from MD simulations at 300 K for monomeric O‐Mad2 (blue line) and O‐Mad2 in a O‐Mad2:C‐Mad2:Mad1^MIM^ dimer (brown dotted line). The continuous error bands indicate one standard deviation derived from six independent simulations. (f) Model of O‐Mad2:C‐Mad2:Mad1^MIM^ based on the O‐Mad2^LL^:C‐Mad2:MBP1^MIM^ structure (PDB 2V64) (Mapelli et al., 2007) highlighting the αC helices at the dimeric interface.

In the physiological context of MCC assembly, O‐Mad2 dimerizes with a Mad1‐bound C‐Mad2 (Lad et al., 2009; Luo et al., 2004; Simonetta et al., 2009; Yang et al., 2008) (Figure 1d). We therefore investigated whether O‐Mad2 undergoes a similar conversion to C‐Mad2 when dimerized with C‐Mad2, as observed for the monomeric O‐Mad2^R133A^ mutant. We first collected HSQC spectra of O‐Mad2^WT^ in its monomeric state (Figure 6a) and then monitored the changes in the spectra upon addition of Cdc20^MIM‐123‐152^ (Figure 6b). O‐Mad2^WT^ adopted a similar global fold to O‐Mad2^R133A^, although with significant CSPs mapping to the β1 strand (Figure S10a,b), and converted to C‐Mad2 upon binding Cdc20^MIM^ (Figure 6b). The resultant C‐Mad2^WT^:Cdc20^MIM^ generated comparable spectra to the dimerization‐deficient C‐Mad2^L13A/R133A^:Cdc20^MIM^ (Figure 6b). Size‐exclusion chromatography coupled with multi‐angle light scattering (SEC‐MALS) data indicated the sample contained a mixture of monomeric and dimeric Mad2 (Figure 6b inset), consistent with the increased NMR linewidths resulting from the higher molecular mass of the dimer. Because Cdc20^MIM^ peptide was added in excess and no O‐Mad2 resonances were observed, we reasoned that the dimer detected in this experiment is a C‐Mad2:C‐Mad2 dimer, similar to that observed previously (Luo et al., 2004; Yang et al., 2008).

We then investigated the O‐Mad2 to C‐Mad2 conversion of O‐Mad2^WT^ in the context of the O‐Mad2^WT^:C‐Mad2^WT^:Mad1^MIM^ dimer. O‐Mad2:C‐Mad2:Mad1^MIM^ was prepared by mixing isotopically labeled O‐Mad2^WT^ with an excess of unlabeled C‐Mad2^WT^ bound to Mad1^MIM^ (Figure 6c). This dimeric species was confirmed by SEC‐MALS (Figure 6c inset). The NMR signature of the Trp side chains of O‐Mad2^WT^ in the context of O‐Mad2:C‐Mad2:Mad1^MIM^ corresponded closely with that of monomeric O‐Mad2^WT^ (Figure S10c), indicating O‐Mad2^WT^ adopted a similar global fold in both monomeric and dimeric states and is consistent with the overall similarity of the solution structure of O‐Mad2^∆NC10^ with the crystal structure of O‐Mad2 in the context of the O‐Mad2^LL^:C‐Mad2:MBP1^MIM^ (Figure S10e) (Luo et al., 2000; Mapelli et al., 2007). Severe line broadening was observed in the spectra of O‐Mad2^WT^:C‐Mad2:Mad1^MIM^, except for the flexible N‐terminus (N10), β5‐αC loop, and β7/β8 hairpin (Figure S10d). This line broadening is attributed to the increased molecular mass of the dimer and conformational changes associated with dimerization. The widespread line broadening across the sequence is comparable to the extensive CSPs in O‐Mad2 upon dimerization with C‐Mad2 (Hara et al., 2015; Mapelli et al., 2006). Similar to monomeric O‐Mad2^WT^, O‐Mad2^WT^ in the O‐Mad2^WT^:C‐Mad2:Mad1^MIM^ dimer converted to C‐Mad2 upon binding Cdc20^MIM^ within 15 min (Figure 6d). The resulting C‐Mad2:Cdc20^MIM^ adopted a similar fold to that of monomeric C‐Mad2^L13A/R133A^:Cdc20^MIM^ (Figure 6d). However, increased linewidths in the spectra indicated the presence of both monomeric and dimeric C‐Mad2, a conclusion further supported by SEC‐MALS analysis (Figure 6d inset).

To assess whether O‐Mad2 dynamics are altered in the O‐Mad2:C‐Mad2:Mad1^MIM^ dimer, we performed MD simulations on the full‐length O‐Mad2:C‐Mad2:Mad1^MIM^ complex, modeled on the crystal structure of O‐Mad2^LL^:C‐Mad2:MBP1^MIM^ (Mapelli et al., 2007). A total of 5.4 μs of simulations were carried out at 300 K. The Cα RMSFs of O‐Mad2 in an O‐Mad2:C‐Mad2 dimer are highly comparable to those of monomeric O‐Mad2 (Figure 6e), except for small decreases at the C‐terminus of the αC helix, located at the dimer interface (Figure 6f). Notably, in contrast to O‐Mad2, MD simulations indicated that the Cα RMSFs of both the β5‐αC loop and residues 160–180 in C‐Mad2 are substantially reduced, being equivalent to Cα residues of the core (Figure S10f). In C‐Mad2, residues 160–180 constitute the “safety‐belt” whereas the β5‐αC loop is restricted by the extended αA helix. The C‐terminus of C‐Mad2 is also substantially less dynamic than that in O‐Mad2, whereas the 10 N‐terminal residues remain highly dynamic in both states (Figure S10f). This agrees with our observation that the N‐terminal residues do not adopt the αN helix in C‐Mad2 in solution (Figure S3d). We conclude that despite the structural differences at the dimeric interface (Figure S10e), O‐Mad2 retains similar structural and dynamic properties upon dimerization and likely undergoes the same O‐Mad2 to C‐Mad2 conversion pathway upon Cdc20^MIM^ binding.

### 2.7 Cdc20^MIM^ promotes the release of the β1 strand

**FIGURE 7:**
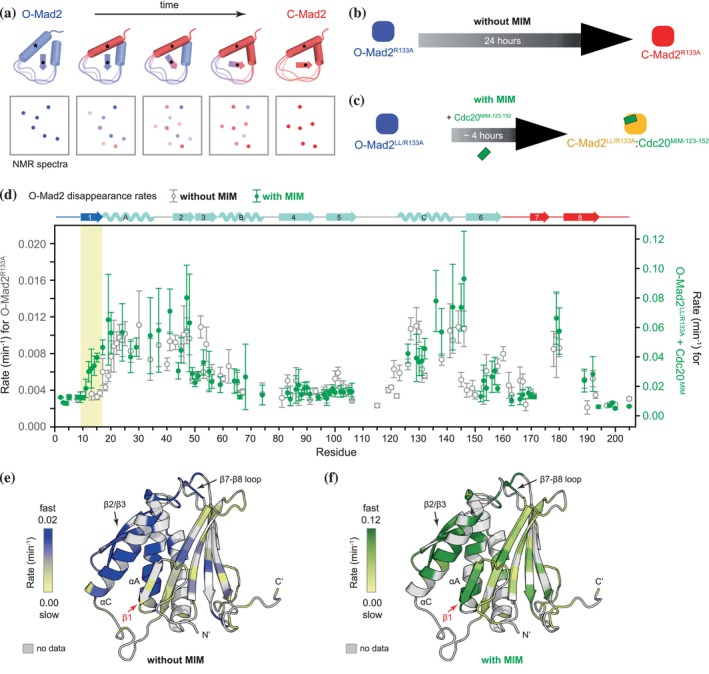
Cdc20^MIM^ promotes the release of the N‐terminal β1 strand. (a) Schematic of O‐ to C‐Mad2 conversion rates using time‐resolved NMR. A series of 2D HSQC spectra were collected at 25°C for 42 h. Disappearance of O‐Mad2 and appearance of C‐Mad2 backbone resonances indicates complete conversion. (b, c) Conversion was measured for O‐Mad2^R133A^ without Cdc20^MIM^ (b) and an O‐Mad2^LL/R133A^ mutant with Cdc20^MIM‐123‐152^, which has reduced conversion (~250 min) (c). (d) The initial conversion rates of individual O‐Mad2 residues were determined by plotting the peak intensities of their O‐Mad2 resonances against time (Figure S11a,b) and fitting the data to an exponential decay curve. Error bars represent the standard regression errors of the fit. The rates for O‐Mad2^R133A^ in the absence of Cdc20^MIM^ were plotted as gray open circles (without Cdc20^MIM^, left y‐axis) and those for O‐Mad2^LL/R133A^ in the presence of Cdc20^MIM‐123‐152^ were plotted as green circles (with Cdc20^MIM^, right y‐axis). The data were scaled for comparison between the varying conversion rates across the sequence. The unscaled data are shown in Figure S11c. Distinct differences are observed for β1, highlighted in yellow. (e, f) The initial conversion rates shown in (d) were mapped onto O‐Mad2 in the absence of Cdc20^MIM^ (e) and in the presence of Cdc20^MIM‐123–152^ (f). The models are colored in a scale, with blue (e) and green (f) showing the highest initial conversion rates. Residues lacking conversion data are colored gray.

The local differences in backbone dynamics (Figure 4) suggested that different structural components in O‐Mad2 might undergo conversion at different rates. To understand the molecular mechanism of the MIM‐induced O‐Mad2 to C‐Mad2 conversion, we recorded the conversion rates of individual residues by monitoring their change in peak intensity in time‐resolved consecutive 2D NMR spectra (Figure 7a). For this experiment, we compared the conversion rates of O‐Mad2^R133A^ in the absence of MIM (Figure 7b) and the O‐Mad2^LL/R133A^ mutant in the presence of Cdc20^MIM‐123‐152^ (Figure 7c). We selected the O‐Mad2^LL/R133A^ mutant for the Cdc20^MIM^‐induced conversion time‐course due to its longer conversion times: ~250 min for complete conversion compared with <15 min for O‐Mad2^R133A^ (Figures 3b and 5). This longer conversion time enabled the acquisition of sufficient NMR spectra to accurately define a time‐course. The disappearance of the backbone resonances from O‐Mad2 and the appearance of the backbone resonances for “empty” C‐Mad2 or C‐Mad2:Cdc20^MIM^ were analyzed by the changes in their peak intensities over 42 h. It was assumed that Mad2 converted from the open to closed state at the end of the measurement and there was no reverse conversion. The disappearance of backbone resonances from O‐Mad2 reflected the transition from the initial open state, whereas the appearance of backbone resonances for C‐Mad2 indicated the complete conversion to the closed state. A direct correlation between the disappearance rates of O‐Mad2 resonances and the appearance rates of C‐Mad2 resonances would indicate the absence of intermediate states on conversion. No global unfolding and refolding were observed during Mad2 conversion, in agreement with proton‐deuterium exchange experiments (Brulotte et al., 2017).

The disappearance of O‐Mad2 resonances reflected the initial conversion rates, with the data fitting closely to an exponential decay curve, both in the presence and absence of Cdc20^MIM^ (coloured blue in Figure S11a,b). Different segments of O‐Mad2^R133A^ showed distinct differences in their O‐Mad2 disappearance rates (Figure 7d). In particular, the αA helix, β2/β3 hairpin, and αC helix exhibited the highest initial conversion rates. Despite the limited data available for the β7/β8 hairpin, signals from the β7‐β8 loop indicated that the β7/β8 hairpin also underwent a similar rate of conversion. O‐Mad2^LL/R133A^ in the presence of Cdc20^MIM^ converted ~8 times faster than O‐Mad2^R133A^ without Cdc20^MIM^ (Figure S11c). However, both shared a mainly similar pattern of varying O‐Mad2 disappearance rates across the sequence (Figure 7d), suggesting O‐Mad2 undergoes a very similar conversion pathway with or without MIM. However, a major difference was observed for the N‐terminal β1 strand (Figure 7d–f). The β1 strand was among the last to convert from its initial state in the absence of Cdc20^MIM^, although at a higher initial conversion rate in the presence of Cdc20^MIM^. This indicated that Cdc20^MIM^ plays a role in triggering the conversion of the β1 strand, most likely by facilitating its release from the core.

Whereas the resonances of O‐Mad2 rapidly disappeared during Mad2 conversion, the resonances of C‐Mad2 slowly appeared during conversion (red and orange in Figure S11a,b). The appearances of C‐Mad2 resonances did not adopt an exponential pattern, unlike the disappearances of O‐Mad2 resonances, and therefore could not be fitted to an exponential plateau curve.

## 3 DISCUSSION

SAC signaling, involving the kinetochore‐catalyzed conformational change of the metamorphic protein Mad2, represents an unusually complex regulatory mechanism. The conformational conversion of O‐Mad2 to C‐Mad2 is the rate‐limiting step in MCC assembly. Whereas the spontaneous Mad2 conversion time in vitro is ~24 h, Cdc20^MIM^ accelerates this conversion by more than 50‐fold (Figure 2c), effectively removing the kinetic energy barrier for MCC formation. Here, based on insights into the dynamics of structural elements of O‐Mad2 and determination of conversion times of individual residues on its transition to C‐Mad2, we propose a model for how Cdc20^MIM^ induces Mad2 remodeling. Our work has implications for understanding the role of MCC‐assembly catalysts, including those of the MCC‐assembly scaffold, in facilitating this function of Cdc20^MIM^.

**FIGURE 8:**
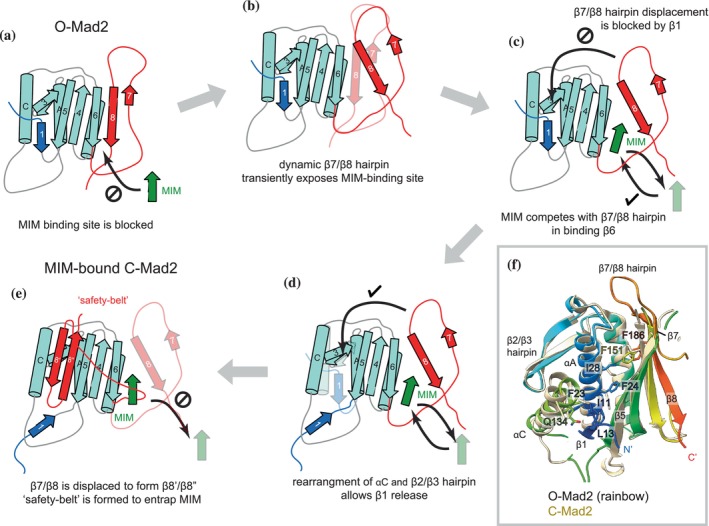
Molecular mechanism of Mad2 conversion. Secondary structure topology diagrams illustrating the suggested molecular mechanisms of the O‐ to C‐Mad2 conversion. (a and e) show the topology of O‐Mad2 and MIM‐bound C‐Mad2, respectively. Residues 1–10 of MIM‐bound C‐Mad2 are represented as unstructured. Displaced β‐strands without hydrogen bonding to the Mad2‐core are expected to be unstructured, but are shown here as β‐strands for illustration. For simplicity, O‐Mad2 is shown as a monomer, but a similar conversion mechanism is expected for O‐Mad2 in an O:C dimer. (b) The β7/β8 hairpin is highly dynamic and transiently unfolds to expose the MIM‐binding site. (c) Cdc20^MIM^ only weakly associates with β6 and competes with the β7/β8 hairpin for binding. (d) Complete remodeling of β7/β8 is blocked by the β1 strand. (e) Cdc20^MIM^ binding to β6 releases the β7/β8 hairpin allowing formation of the β8′/β8″ hairpin that competes with β1 for pairing with β5. The reconfigured C‐terminus of C‐Mad2 entraps Cdc20^MIM^ through a “safety‐belt” connecting β6 with β8′. The transient Mad2:Cdc20^MIM^ intermediate has lower free energy than the “empty” Mad2 equivalent, reducing the energy barrier to C‐Mad2 formation. (f) Superimposition of O‐Mad2 (N‐ to C‐termini colored blue‐to‐red) onto C‐Mad2 (straw) (from O‐Mad2:C‐Mad2:MBP1^MIM^ (Mapelli et al., 2007) illustrating the network of hydrophobic residues linking Phe186 of β8 to Ile11 and Leu13 of β1 through Phe151 of β5, Ile28, Phe24, and Phe23 of αA. Side chains of these residues reposition, as does αC, on the O‐to‐C‐Mad2 conversion.

Our data confirm that the MIM‐binding site is not accessible in O‐Mad2 (Figures 3 and 8a), in agreement with previous observations that Mad2 variants trapped in the open state do not bind Cdc20^MIM^ (De Antoni et al., 2005; Luo et al., 2000; Mapelli et al., 2007; Yang et al., 2008). For binding to occur, O‐Mad2 must undergo a conformational conversion to expose the MIM‐binding site that is obstructed by the β7/β8 hairpin. NMR dynamics data and MD simulations are consistent with the idea that the β7/β8 hairpin is a highly flexible structural segment of O‐Mad2, suggesting that it may transiently unfold to expose the MIM‐binding site (Figures 4 and 8b). We showed that Cdc20^MIM^ binds with low affinity to a mutant of Mad2 lacking the β7/β8 hairpin (O‐Mad2^∆β7/β8^) (Figure 3g), in agreement with a previous study (Yang et al., 2008). Since O‐Mad2^∆β7/β8^ is a structural mimic of O‐Mad2 with a displaced β7/β8 hairpin, a likely crucial early event in the O‐ to C‐Mad2 conversion is the displacement of the β7/β8 hairpin and pairing of Cdc20^MIM^ to β6, as previously proposed by Musacchio and colleagues (Piano et al., 2021). The low affinity of this interaction necessitates that Cdc20^MIM^ is positioned close and in the correct relative orientation to its binding site on Mad2, a function fulfilled by the MCC‐assembly scaffold that serves to increase the local concentrations of Cdc20^MIM^ and O‐Mad2 (Fischer et al., 2022). It also suggests that Cdc20^MIM^ is only locked in place in C‐Mad2 by the “safety‐belt” created from a restructured β6‐β7 loop, which is secured by the pairing of the β8′/β8” hairpin with β5 of the Mad2 core β sheet (Piano et al., 2021). The β8′/β8″ hairpin in C‐Mad2 is comprised of residues that form the β7/β8 hairpin and most of the flexible C‐terminus of O‐Mad2 (Figure 1b,c). Pairing of the β8′/β8″ hairpin with β5 requires displacement of β1 and its remodeling to form the N‐terminus of αA. Without release of the β1 strand, the β7/β8 hairpin cannot be stably displaced and remodeled as β8′/β8″ and would compete with Cdc20^MIM^ for the MIM‐binding site on β6 (Figure 8c). Remodeling of β1 is slowed in the O‐Mad2^LL^ mutant because the shortened β5‐αC loop impedes the passage of β1‐strand residues through the β5‐αC loop, thereby restricting their access to the N‐terminus of αA. In contrast, destabilizing β1 as caused by the Mad2^L13A^ mutant, or its complete truncation, as in Mad2^∆N15^, traps Mad2 in a closed conformation (Mapelli et al., 2006; Mapelli et al., 2007; Yang et al., 2007). Thus, factors that promote β1 strand release and its remodeling would contribute to accelerating the O‐Mad2 to C‐Mad2 conversion.

In this study, we provide NMR evidence that Cdc20^MIM^ promotes the structural conversion of β1‐strand residues from their O‐Mad2 conformation (Figure 7). This reveals a link between Cdc20^MIM^ binding and β1 strand displacement. One explanation for this is a potential allosteric coupling of the β7/β8 hairpin and β1 strand release mediated through hydrophobic side chains on the β6 strand and αA and αC helices that link β7/β8 with β1 (Figure 8f) (Mapelli et al., 2007). These conformational changes associated with the O‐Mad2 to C‐Mad2 conversion might be triggered by a loss of hydrophobic contacts between Phe186 of β8 and Phe151 of β6 caused by β7/β8 displacement. Phe151 in turn contacts the cluster of αA helix residues Phe23, Phe24, and Ile28. Rotation of the aromatic side chains of Phe23 and Phe24 blocks the binding pocket for Leu13 of β1 and is also linked to a shift of αC that contacts Ile11 of β1 (Figure 8f). The rearrangement of aromatic residues is consistent with the distinctly different NMR fingerprint spectra for O‐Mad2 and C‐Mad2 (Figure S2d,e). Another factor linking Cdc20^MIM^ to β1 strand displacement is that Cdc20^MIM^ and the β7/β8 hairpin compete for binding to the β6 strand (Figure 8c). With Cdc20^MIM^ bound to β6, residues of the displaced β7/β8 hairpin would be more likely to compete with β1 to pair with β5 (Figure 8d,e). Transient displacement of the β7/β8 hairpin allows Cdc20^MIM^ binding and lowers the free energy state of this MIM‐bound intermediate (relative to non‐bound). Stabilization and formation of the newly folded C‐Mad2 β8′/β8″ hairpin and N‐terminal turn of the αA helix are likely cooperative and coordinated. Hydrogen bonds linking Ser16 of αA with Thr188 and His191 of β8′/β8″ stabilize C‐Mad2 (Yang et al., 2008; Ye et al., 2017), whereas the disruption of these hydrogen bonds was proposed to contribute to the ATP‐dependent TRIP13‐p31^comet^ catalyzed remodeling of C‐Mad2 into O‐Mad2 (Alfieri et al., 2018). The lack of a direct correlation between the O‐Mad2 disappearance rates and C‐Mad2 appearance rates (Figure 7d) suggests the presence of intermediate states during the O‐Mad2 to C‐Mad2 conversion. This would be consistent with an O‐Mad2 to C‐Mad2 conversion model in which O‐Mad2 undergoes a partial conversion involving the displacement of β7/β8 hairpin prior to the release of the β1‐strand.

Finally, the self‐association of O‐Mad2 with C‐Mad2 modestly contributes to catalyzing the O‐ to C‐Mad2 conversion, as originally proposed in the Mad2 template model (De Antoni et al., 2005) and demonstrated experimentally (De Antoni et al., 2005; Faesen et al., 2017; Kulukian et al., 2009; Lad et al., 2009; Nezi et al., 2006; Simonetta et al., 2009). As Mad2 dimerization led to lower sensitivity and longer acquisition times in NMR measurements, we were unable to use NMR to demonstrate that the dimerization of O‐Mad2 with C‐Mad2 increases conversion rates induced by Cdc20^MIM^. Two previous studies reported differences in the HSQC spectra between monomeric O‐Mad2 and O‐Mad2 in the context of the O‐Mad2:C‐Mad2 dimer (Hara et al., 2015; Mapelli et al., 2006), indicative of dimer‐induced structural changes mainly localized to interfacial residues of the αC helix and β2/β3 hairpin (Hara et al., 2015). O‐Mad2 dimerization with C‐Mad2 may therefore induce structural changes at the β5/αC cleft of O‐Mad2, contributing to destabilizing β1 and explaining how dimerization contributes to catalyzing the O‐Mad2 to C‐Mad2 conversion.

The capacity of Cdc20^MIM^ peptides to greatly accelerate the intrinsically slow O‐Mad2 to C‐Mad2 conversion provides mechanistic insight into how unattached kinetochores activate the SAC (Rieder et al., 1995). SAC signaling is mediated by the MCC (Sudakin et al., 2001), whose assembly is rate‐limited by the formation of C‐Mad2:Cdc20. SAC catalysts (Faesen et al., 2017; Ji et al., 2017; Piano et al., 2021) generate an MCC‐assembly scaffold that functions to present the exposed Cdc20^MIM^ to its binding site on Mad2 (Chen et al., 2023; Fischer et al., 2022; Ji et al., 2017; Lara‐Gonzalez et al., 2021; Piano et al., 2021). In contrast to the rapid association of Cdc20^MIM^ peptides to empty C‐Mad2, full‐length Cdc20 does not readily bind to empty C‐Mad2 (Piano et al., 2021), likely due to the steric hindrance of threading the long N‐terminal segment of Cdc20 through the closed safety‐belt of C‐Mad2 (Piano et al., 2021). Thus, an inherent feature of SAC activation, rapid formation of C‐Mad2, is coordinated with the presentation of its ligand, Cdc20^MIM^. This is achieved by the exposure of Cdc20^MIM^ through the relief of a Cdc20‐auto‐inhibitory mechanism, combined with its close spatial positioning to its cryptic binding site on O‐Mad2. Because empty C‐Mad2 is non‐productive for Cdc20 binding (Piano et al., 2021), the Cdc20^MIM^‐induced triggering of the O‐Mad2 to C‐Mad2 conversion is an elegant mechanism to regulate MCC assembly and thus SAC signaling.

Understanding how Cdc20^MIM^ directly induces the O‐Mad2 to C‐Mad2 conversion is essential to our understanding of how MCC assembles. However, the intrinsic nature of O‐Mad2 to undergo conversion to C‐Mad2 has made it difficult to fully characterize this protein. Building on decades of structural work on Mad2, our study provides new insights into the dynamics of O‐Mad2, demonstrating how protein dynamics coupled to Cdc20^MIM^ binding play a key mechanistic role in accelerating the Mad2 conversion rate and serve as a framework for understanding the mechanisms of conformational conversion of metamorphic proteins.

## 4 MATERIALS AND METHODS

### 4.1 Cloning Mad2 expression vectors

The cloning of human wild‐type Mad2 and the mutant Mad2^R133A^ by the USER® method (NEB) into the pRSFDuet‐1 vector (71341‐3, Sigma‐Aldrich) with a His_6_‐DoubleStrep‐TEV tag at its N‐terminus was described previously (Fischer et al., 2022). For this study, Mad2 mutants were generated as follows. The Mad2^LL^ expression construct was generated from the wild‐type Mad2 expression vector (Fischer et al., 2022) by replacing residues 109–117 (TAKDDSAPR) with GSG. Mad2^ΔC10^ and Mad2^Δβ7/β8^ were modified from wild‐type Mad2 expression construct by deleting residues 196–205 and 166–205, respectively. Mad2^LL/R133A^ was modified by substituting Ala for Arg133 from the Mad2^LL^ expression construct. Mad2^LL/ΔC10^ was modified from Mad2^LL^ by deleting residues 196–205. Mad2^L13A/R133A^ was generated from the Mad2^R133A^ mutant. Site‐directed mutagenesis of Mad2 was completed using the QuikChangeTM Lightning Site‐Directed Mutagenesis Kit (Agilent) developed by Stratagene Inc. (La Jolla, CA). A schematic of Mad2 variants is shown in Figure 1e.

### 4.2 Protein expression and purification

Mad2 constructs were expressed in BL21 Star (DE3) cells (Thermo Fisher Scientific), co‐transformed with a pRARE plasmid (Merck). Uniformly labeled proteins were expressed in M9 minimal media (6 g/L Na_2_HPO_4_, 3 g/L KH_2_PO_4_, 0.5 g/L NaCl) supplemented with 1.7 g/L yeast nitrogen base without NH_4_Cl and amino acids (Sigma Y1251). 1 g/L ^15^NH_4_Cl and 4 g/L glucose were supplemented for ^15^N labeling. For ^13^C/^15^N double‐labeled samples, unlabeled glucose was replaced with 3 g/L ^13^C‐glucose. Protein expression was induced with 0.4 mM IPTG at 16°C overnight. All samples were purified as previously described (Luo & Yu, 2005; Mapelli et al., 2007) and all O‐Mad2 variants were kept at 4°C during purification to prevent conversion to C‐Mad2. Prior to all NMR experiments, proteins were dialyzed into 20 mM HEPES, pH 7.0, 100 mM NaCl, 1 mM TCEP. ^13^C/^15^N‐labeled samples were used for backbone assignment, and all other experimental data were acquired on ^15^N‐labeled samples. Proteins were kept on ice before NMR experiments to ensure all O‐Mad2 remained in the open state. For “bound” C‐Mad2, MIM peptides were added in 1:2 molar excess to ensure complete conversion.

For wild‐type Mad2, the protein was expressed as O‐Mad2^WT^ using the conditions described above. C‐Mad2^WT^ was generated by adding an excess of Mad1^MIM^ peptide to O‐Mad2^WT^ and the sample was further purified using size‐exclusion chromatography (SEC), where only monomeric C‐Mad2^WT^:Mad1^MIM^ was used for analysis. The dimeric O‐Mad2^WT^:C‐Mad2^WT^:Mad1^MIM^ sample was prepared by mixing isotopically labeled O‐Mad2^WT^ with a two‐fold molar excess of unlabeled C‐Mad2^WT^:Mad1^MIM^. The sample was further purified using SEC, where only dimeric O:Mad2^WT^:C‐Mad2^WT^:Mad1^MIM^ was used for analysis.

### 4.3 Peptide synthesis

All peptides were synthesized by Cambridge Research Biochemicals, UK. Peptides were dialyzed into 20 mM HEPES, pH 7.0, 100 mM NaCl, and 1 mM TCEP for NMR experiments. The sequences for the peptides used in this study are: Cdc20^MIM‐111‐138^ (EHQKAWALNLNGFDVEEAKILRLSGKPQ), Cdc20^MIM‐123‐137^ (FDVEEAKILRLSGKP), Cdc20^MIM‐123‐152^ (FDVEEAKILRLSGKPQNAPEGYQNRLKVLY), Cdc20^MIM‐138‐152^ (QNAPEGYQNRLKVLY), Mad1^MIM‐529‐550^ (RALQGDYDQSRTKVLHMSLNPT), and MBP1^MIM^ (SWYSYPPPQRAV).

### 4.4 Sample conditions for NMR

All NMR data on Mad2 were acquired in a 5 mm tube at 25°C in 20 mM HEPES, pH 7.0, 100 mM NaCl, 1 mM TCEP, 5% D_2_O. Backbone assignment experiments and all spectra, unless otherwise denoted, were collected using an in‐house Bruker 800 MHz Avance III spectrometer, equipped with a triple resonance TCI CryoProbe. 2D spectra were acquired using BEST ^1^H,^15^N‐TROSY HSQC, with 16 scans and a recycle delay of 400 ms, giving an experimental time of 12 min per spectrum. A Bruker 950 MHz Avance III spectrometer (MRC Biomedical NMR Centre, Francis Crick Institute) was used for the residue conversion rate analysis and the characterization of Mad2 dimers. Most experiments, including all backbone experiments, were acquired with 100 μM samples; the residue‐specific conversion rate analysis and characterization of Mad2 dimers utilized 200 μM samples and 300 μM samples, respectively.

### 4.5 Backbone assignment and secondary structure analysis

Backbone resonance assignments of multiple conformers of Mad2 were based on the following triple resonance experiments acquired with ^13^C,^15^N labeled samples: HNCO, HN(CA)CO, HNCA, HN(CO)CA, HNCACB, CBCA(CO)NH (Bruker pulse sequence library). All 3D datasets were collected with 15–30% non‐uniform sampling and processed in MddNMR (Jaravine et al., 2008) using compressed sensing reconstruction. Backbone resonances were assigned in Sparky3 (T.D. Goddard and D.G. Kneller, UCSF) supported by in‐house scripts and Mars (Jung & Zweckstetter, 2004). 2D ^1^H,^1^H NOESY spectra were recorded with mixing times of 100 ms to assign the indole NH resonances of the tryptophan side chains. Topspin 4.1.1 (Bruker) was used for processing and NMRFAM‐Sparky 1.47 for data analysis (Lee et al., 2015).

Backbone assignment for full‐length O‐Mad2 was achieved using a combination of four variants, Mad2^R133A^ (a dimerization‐deficient mutant, Sironi et al., 2002), Mad2^LL^ (Mapelli et al., 2007), Mad2^∆C10^, and Mad2^LL/∆C10^. Mad2^∆C10^ and Mad2^LL/∆C10^ provide the long‐term stability required for NMR triple resonance experiments. The backbone resonances for the missing β5‐αC loop in Mad2^LL^ were assigned using Mad2^∆C10^. The backbone resonances for the C‐terminal residues were assigned using a limited set of Cα chemical shifts from Mad2^LL^, as Mad2^LL^ also has a limited stability of <3 days and undergoes slow O‐Mad2 to‐C Mad2 conversion. The HSQC spectra of these variants overlapped closely, indicating that the global folds of these mutants are similar (Figure S6a). All the assignments were consolidated and transferred to full‐length Mad2^R133A^ (Figure S2a). Backbone resonances for “empty” C‐Mad2 were assigned using the double mutant Mad2^L13A/R133A^ (Yang et al., 2008) (Figure S2b). Backbone resonances of C‐Mad2:Cdc20^MIM^ were assigned using full‐length Mad2^L13A/R133A^ in the presence of a two‐fold molar excess of Cdc20^MIM^, spanning residues 111–138 (Cdc20^MIM‐111‐138^) (Figure S2c). Of the 199 non‐proline residues in Mad2, the backbone amide resonances of 177 residues were assigned for O‐Mad2, 163 residues were assigned for “empty” C‐Mad2, and 177 residues for C‐Mad2:Cdc20^MIM^. Tryptophan side chain resonances shown in Figure 2a were assigned from ^1^H,^1^H NOESY experiments (O‐Mad2, C‐Mad2, C‐Mad2:Cdc20^MIM^) or taken from the BMRB database (4775 for O‐Mad2 and 5299 for C‐Mad2:MBP1^MIM^). The combined data allowed the mapping of tryptophan side chain chemical shifts in the experimentally determined structures (PDB 1DUJ, 1S2H, 6TLJ, 1GO4, and 1KLQ) (Alfieri et al., 2016; Alfieri et al., 2020; Luo et al., 2000; Luo et al., 2002; Luo et al., 2004; Sironi et al., 2002).

Secondary chemical shifts were calculated using the equation (δCɑ_obs_ – δCɑ_rc_) – (δCβ_obs_ – δCβ_rc_) where δCɑ_obs_ and δCβ_obs_ are the observed Cɑ and Cβ chemical shifts, and δCɑ_rc_ and δCβ_rc_ are the Cɑ and Cβ chemical shifts for random coils (Kakeshpour et al., 2021; Spera & Bax, 1991). Random coil chemical shifts were calculated based on sequence using POTENCI (Nielsen & Mulder, 2018), taking into account the effects of pH, temperature, and buffer pKa.

### 4.6 CSP analysis

Weighted CSPs were calculated using the equation (Williamson, 2013): Δδ = [((Δδ_HN_W_HN_)^2^ + ((Δδ_N_W_N_)^2^)^2^]^1/2^, where Δδ^1^H and Δδ^15^N are the CSPs in ^1^H and ^15^N dimensions, respectively (Ayed et al., 2001). The weight factors were determined from the average variances of chemical shifts in the BMRB database (Mulder et al., 1999), with W_HN_ = 1 and W_N_ = 0.16. Binding affinities K_D_ were estimated from the residues with the most significant CSP. CSPs were plotted against the ligand concentration and fitted to the following equation: Δδ_obs_ = Δδ_max_ [(P + L + K_D_) – (P + L + K_D_)^2^ – 4PL]^1/2^/2P (Williamson, 2013) where P and L are the total concentrations of protein and ligand, respectively; Δδ_obs_ is the observed CSP and Δδ_max_ is the maximum CSP upon saturation.

### 4.7 Relaxation measurements

^15^N T_2_ relaxation times of O‐Mad2^∆C10^ were measured at 950 MHz proton resonance frequency using INEPT‐based pseudo‐3D CPMG pulse sequences with a recovery delay of 5 s incorporating a temperature‐compensation scheme (hsqct2etf3gpsitc3d, Bruker). Twelve mixing times were collected in an interleaved fashion to avoid bias due to the slow interconversion (7.84, 15.68, 31.36, 47.04, 62.72, 78.4, 94.08, 109.76, 125.44, 141.12, 156.8, and a repeat 7.84 ms). Peak intensities were fitted to an exponential decay curve in NMRFAM‐Sparky 1.47 (Lee et al., 2015).

^15^N T_1_ relaxation times of O‐Mad2^∆C10^ were measured at 950 MHz proton resonance frequency using INEPT‐based pseudo‐3D pulse sequences with a recovery delay of 5 s incorporating a temperature compensation scheme (hsqct1etf3gpsitc3d, Bruker). Twelve interleaved mixing times were collected (0.01, 0.02, 0.04, 0.08, 0.12, 0.16, 0.32, 0.64, 0.96, 1.28, 1.60, and a repeat 0.01 s). Peak intensities were fitted to an exponential decay curve in NMRFAM‐Sparky 1.47 (Lee et al., 2015).

To maximize sensitivity and resolution, dynamics data were acquired at higher field strength corresponding to proton resonance frequencies of 950 MHz.

### 4.8 SEC‐MALS

Samples were analyzed using a Heleos II 18‐angle light scattering instrument (Wyatt Technology) and an Optilab rEX online refractive index detector (Wyatt Technology) at 4 °C. A 100 μL Mad2 sample at 300 μM was loaded onto a Superdex 75 10/300 GL increase column (GE Healthcare) pre‐equilibrated with 20 mM HEPES, pH 7.0, 100 mM NaCl, 1 mM TCEP, 5% D_2_O, running at 0.5 mL/min. The molecular mass was determined from the intercept of the Debye plot using the Zimm model as implemented in the Astra software (Wyatt Technology). Protein concentration was determined from the excess differential refractive index based on a 0.186 refractive index increment for a 1 g/mL protein solution.

### 4.9 Analysis of Mad2‐residue conversion rates

Consecutive series of ^1^H,^15^N SOFAST‐HMQC experiments were acquired in a pseudo‐3D fashion to ensure all spectra were collected in near‐identical conditions. SOFAST‐HMQC was chosen for its short pulse sequence to maximize the number of data points collected during Mad2 conversion. All parameters were set up using a test sample to minimize the dead time in data acquisition. Each 2D spectrum was acquired with eight scans and a recycle delay of 200 ms and 256 complex points in the indirect dimension, giving a final spectral resolution of 2.8 Hz per point in the indirect dimension and an experimental time of 5 min per spectrum. A total of 500 spectra were collected to monitor Mad2 conversion over 42 h. All spectra were processed using in‐house scripts and NMRPipe (Delaglio et al., 1995). Four spectra were sequentially added to enhance signal‐to‐noise, and consecutive data points have a 5‐min offset. Peak intensities for each added spectrum were analyzed using Poky build:20230213 (Lee et al., 2021) and extracted using a modified Python module. Peak intensities for O‐Mad2 were normalized with the initial peak intensities in the first added spectrum, assuming all Mad2 molecules were in the open state at the initial time point. Peak intensities for C‐Mad2 were normalized with the final peak intensities in the last added spectrum. It was assumed that all Mad2 proteins were converted from open to close at the end of the measurement. We also assumed there was no reverse conversion of C‐Mad2 to O‐Mad2, as this is an ATP‐dependent process catalyzed by the AAA+ ATPase TRIP13 in combination with the p31^comet^ adaptor protein (Eytan et al., 2014; Teichner et al., 2011). For the disappearance of O‐Mad2 resonances, data were fitted to an exponential decay curve with nonlinear regression, using the equation: y = (y_0_ – y_min_) exp (−*k*x) + y_min_, where y_0_ is the initial peak intensity, y_m_ is the minimum peak intensity, and *k* is the rate constant. For the appearance of C‐Mad2 resonances, the data were not fitted as they do not show a clear exponential or delayed exponential increase. Peaks that are overlapping in the O‐Mad2 and C‐Mad2 spectra were discarded in the analysis. Normalization of peak intensities, curve fitting, and plotting were carried out using in‐house Python scripts.

### 4.10 MD simulations

The starting structure for MD simulations on monomeric full‐length O‐Mad2 was built using the crystal structure of O‐Mad2^LL^ (PDB 2 V64, Mapelli et al., 2007, chain D) and the missing segments (residues 1–8, 109–118, 195–205) were built using Modeller (Webb & Sali, 2016). The C‐terminal 10 residues were manually truncated for the simulation of Mad2^∆C10^. The starting structure for MD simulations on full‐length O‐Mad2:C‐Mad2:Mad1^MIM^ was built using the crystal structure of O‐Mad2^LL^:C‐Mad2:MBP1^MIM^ (PDB 2V64, Mapelli et al., 2007). The missing segments in O‐Mad2 were built as previously described, and MBP1^MIM^ was manually modified to match the sequence of Mad1^MIM‐538‐551^ (SRTKVLHMSLNPTS) in PyMOL (Schrödinger, LLC), guided by the structure of C‐Mad2^R133A^:Mad1^485‐718^ (PDB 1GO4 (Sironi et al., 2002)). Cys79 and Cys106 were defined as unoxidized. The O‐Mad2 system had 67,979 atoms, and the O‐Mad2:C‐Mad2:Mad1^MIM^ system had 158,552 atoms. 100 mM NaCl was added to the solvent to match the NMR conditions. Simulations were carried out using the DES‐Amber force field and TIP4P‐D water model (Piana et al., 2020). The DES‐Amber force field was chosen for its suitability to study proteins with both folded and disordered regions. MD simulations were carried out in GROMACS (Abraham et al., 2015) with the Verlet leapfrog integrator, a 2 fs time step, constrained bonds to hydrogen, Particle Mesh Ewald for long‐range electrostatics, and a 1 nm cut‐off for non‐bonded interactions. A steepest descent energy minimization protocol was used. Cubic boundaries with a 1 nm distance between the protein and the boundary were used, and water was added to the box. The V‐rescale thermostat and Parrinello–Rahman barostat were used to maintain a reference temperature/pressure. A 100 ps NVT equilibration at the target temperature and a 100 ps NPT equilibration preceded 0.9 μs production runs in the NPT ensemble, with snapshots saved every 200 ps. A total of 5.4 μs of simulations were carried out for each Mad2 variant or at each temperature, using six independent simulations of 0.9 μs. For each snapshot, the RMSD was calculated for all protein backbone atoms to the starting structure. Cα RMSF was calculated by fitting each snapshot to the average coordinates, calculating the mean squared distance to the average coordinates across snapshots for each Cα, and averaging over the simulation repeats using six independent simulations of 0.9 μs with different starting velocities. Both RMSD and RMSF were calculated using standard GROMACS (Abraham et al., 2015) scripts and plotted using GraphPad PRISM 9.5.1.

For the simulation of Mad2^∆β7/β8^ in the presence of the MIM peptide, the β7/β8 residues were manually truncated and a single MIM peptide (FDVEEAKILRLSGKPQNAPEGYQNRLKVLY) was added to the 9 nm cubic simulation box. A total of 12 μs of simulation was carried out using six independent simulations of 2 μs with the MIM peptide starting conformation in each determined by a short implicit solvent simulation (Greener, 2024). For analysis, the first 200 ns of each simulation was excluded, and the contact probability for each residue was the fraction of snapshots where the Cβ atom (Cα for glycine) was closer than 8 Å to any Cβ in the MIM peptide.

### 4.11 Other computational methods

Molecular graphics were produced in PyMOL Molecular Graphics System, Version 2.5.3, Schrödinger, LLC. Secondary structure topology diagrams were drawn with Biotite (Kunzmann & Hamacher, 2018) and CCP4 Topdraw (Bond, 2003), using their structural annotations from the NCBI database.

## AUTHOR CONTRIBUTIONS

**Conny W. H. Yu:** Conceptualization; investigation; visualization; methodology; writing – original draft; formal analysis. **Elyse S. Fischer:** Investigation; conceptualization; writing – review and editing. **Joe G. Greener:** Conceptualization; investigation; writing – review and editing; formal analysis. **Jing Yang:** Investigation. **Ziguo Zhang:** Investigation. **Stefan M. V. Freund:** Investigation; writing – review and editing; conceptualization; formal analysis. **David Barford:** Conceptualization; investigation; funding acquisition; visualization; supervision; resources; project administration; writing – review and editing; writing – original draft; formal analysis.

## CONFLICT OF INTEREST STATEMENT

The authors declare no competing interests.

## Supporting information


**Data S1:** Supplementary Figures

## Data Availability

The data that support the findings of this study are available from the corresponding author upon reasonable request.
